# Evaluation of selected minerals and health risk and proximate analysis of wasawasa (a street food)

**DOI:** 10.1016/j.toxrep.2024.101785

**Published:** 2024-10-22

**Authors:** Marian Asantewah Nkansah, Fati Haruna, Dominic Adrewie

**Affiliations:** Department of Chemistry, Kwame Nkrumah University of Science and Technology, Kumasi, Ghana

**Keywords:** Heavy metals, Proximate analysis, Wasawasa (a street food), Health risk, Yam peels

## Abstract

This study was carried out to determine the proximate composition and the potential heavy metal health risk that may be associated with the consumption of wasawasa, a dish made from locally milled yam peels, by examining the presence of six metals (iron, nickel, chromium, sodium, and magnesium and potassium) in samples procured. Sixteen (16) samples of ready-to-eat wasawasa were collected from Aboabo, Manhyia, Sawaba, Asawase, Adenyase, and Ayigya in Kumasi, since these are the communities where wasawasa is mainly produced, sold, and consumed. The samples were digested with a nitric, perchloric, and sulfuric acid mixture and analyzed using a microwave plasma atomic emission spectrometer (Agilent 4210 MP-AES). The average concentrations of metals were Na (8506.88 mg/kg), Mg (222.63 mg/kg), Fe (84.45 mg/kg), Cr (2.31 mg/kg), K (1702.08 mg/kg, and Ni (1.12 mg/kg). Proximate analysis was used to determine Protein, fat, ash, moisture, and fiber content of the local wasawasa, which were found to be 15.667 %, 0.45 %, 1.00 %, 27.54 %, and 0.41 %, respectively. The hazard index of the heavy metals (Fe, Ni and Cr) for both adults and children were each greater than one, indicating the population is likely to experience non-carcinogenic effects from the consumption of wasawasa.

## Graphical Abstract

**Figure:**
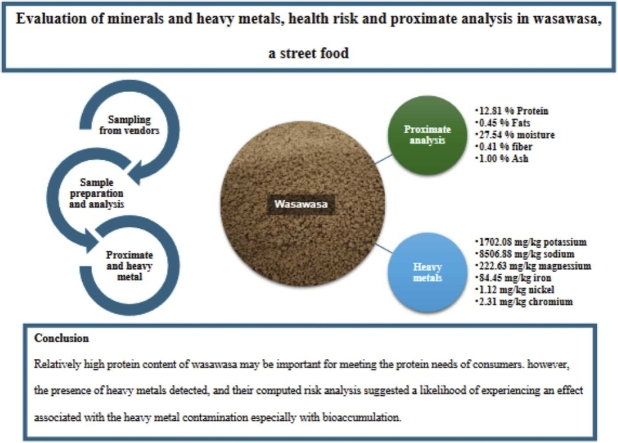

## 1 Introduction

Yam, the common name for the *Dioscorea* Genus with over 600 species [1]. It is a perennial herbaceous vine cultivated for the consumption of their starchy tubers and for medicinal purposes in many temperate and tropical regions, especially in West Africa, South America and the Caribbean, Asia, and Oceania [1], [2].

Yam, considered to be nutritionally superior to other tubers [3], contains minerals such as phosphorus, calcium, and iron, as well as sodium and magnesium. It is also a good source of carbohydrates and energy but a very poor source of fat [4]. The nutritional composition of yam is affected by the species or cultivar, the mode of processing and cooking, as well as the duration of the preparation [5], [6]. White Guinea yam, *Dioscorea rotundata*, is one of the most important and dominant species in West and Central Africa yam production zones. Secondary metabolites such as oxalates, tannins, and phytates have also been found in yam, typically in amounts that are safe for food processing applications [5], [7]. Some popular foods prepared from yam include fufu, ampesia, petepete, yam rice for children, and wasawasa.

Yam processing methods include boiling, drying, milling into flour, peeling, and roasting. Farmers commercially cultivate mainly *Dioscorea rotundata* (26 varieties) and, to a lesser extent, *D*. *alata* (13 varieties). With US$ 48.2 million in yam exports in 2021, Ghana became the world's biggest yam exporter during the year under study, with the USA accounting for 21.9 % of Ghana's yam sales in 2021 [8].

Since the industrial revolution and economic globalization, the diversity of environmental contaminants has increased exponentially, with countless anthropogenic sources. [9]. Heavy metals are elements with a density greater than 5 g/cm [10]. Some heavy metals like iron, zinc, and copper are essential for the body in minute amounts as components of metabolic processes, while others, such as cadmium, lead, and arsenic, are cumulative poisons capable of causing environmental hazards and are reported to be exceptionally toxic [11]. Due to their bioaccumulation and non-biodegradability, these metals contaminate the food chain and subsequently become a source of toxicity for human beings and the entire ecological system.[12]. Several communities in Ghana have experienced direct or indirect heavy metal pollution from agricultural and industrial practices [13]. Due to modernization, many consumers are more interested in convenience and thus pay little attention to the hygiene, safety, and quality of street-vended foods. There is therefore a need to determine the concentration of heavy metals in Wasawasa and the long-term health risks the population might be exposed to. Wasawasa is a street food mostly eaten in the northern regions of Ghana and it has slowly found its way into some southern parts of the country, mostly through rural urban migration. Made from dried, milled yam peels, the dish is a dark or black color and is served with vegetables and fish. The dried yam peels are milled at local mills, popularly known as “Nika Nika”. The metallic disks used in the mills are mostly made in Ghana from grey cast iron by local foundries with little or no chemical knowledge about metals[14]. Some are also imported from China and India, although local ones are preferred due to affordability. Studies have indicated the leaching of heavy metals like iron, lead, copper, chromium, cadmium, nickel, and zinc into milled food [15]. Heavy metals have been reported in yam tubers [16], [17], yam flour [18], groundnut paste [19], milled shrimp [20] milled maize flour [21] and milled fufu [22] in Ghana. This study was carried out to determine the proximate composition and potential metal health risk that may be associated with the consumption of wasawasa.

## 2 Materials and methods

### 2.1 Study area

The study was carried out in six (6) communities in the Ashanti regional capital, Kumasi. Kumasi is the commercial, industrial, and cultural capital of the historical Ashanti empire. With a bustling population accounting for about 17.7 percent of the total population of Ghana in 2021 as reported by the Ghana Statistical Service [23]. It lies between longitude 1.300 and 1.350 and latitude 6.35 and 6.400 with an elevation of 200–250 above sea level. The annual temperature and rainfall of the city are 30.7 °C (87.3 °F) and 1400 mm, respectively. The city also houses the largest market in West Africa (Kejetia), which has contributed to the influx of rural migrants in search of work and greener pastures.

## 3 Sampling

A total of sixteen (16) ready to eat wasawasa were sampled from six (6) communities namely: Manhyia, Aboabo, Sawaba, Asawase, Adukrom, and Adenyase in the Kumasi metropolis. Each sample was packaged in labelled zip-lock bags before transported to the laboratory for analysis.

## 4 Sample preparation

Wet acid digestion was employed to isolate the metals in the food samples from their matrices before assessing them with atomic emission spectroscopy (AES). For each sample, 1.0 g was carefully taken using a digital balance. Each 1.0 g of food sample was digested by heating with a digestion mixture of 10 ml concentrated nitric and perchloric acid (1:1) and 5 ml sulfuric acid in a 50 ml digestion tube. Each sample mixture was evaporated on a hot plate in a fume hood at 2000 °C for forty (40) minutes until the brown fumes disappeared, leaving a clear digest. The digestion of a reagent blank was carried out in parallel with the wasawasa samples, with the same digestion parameters. The clear digests were cooled, filtered, and diluted with distilled water to the 50 ml mark.

### 4.1 Mineral analysis

The mineral composition was determined in each digest using spectrophotometry. A microwave-plasma atomic emission spectrophotometer (MP-AES) (Model Agilent 4210 MP-AES, US) was used to evaluate the materials. The data were read in triplicate and expressed in milligram per kilogram (mg/kg).

### 4.2 Statistical analysis and health risk indices

Statistical analysis such as average mineral and heavy metal concentration and Pearson’s correlation analysis were conducted on the results with the help of OriginPro and Microsoft Office excel. The estimated daily intake (EDI), Hazard quotients (HQ) and hazard index (HI) were determined using the respective equations: EDI = (M] ×M)/W, HQ = EDI/Rf and HI = ∑HQ. Where [M] is concentration of the metal, M (0.10 or 0.20 kg) is the mass of groundnut paste consumed each day by an adult or child and W (70 or 15 kg) is the average weight of a normal adult or child respectively. The oral reference doses (Rf) for Fe, Ni, and Cr are 0.007, 0.02, and 0.003 mg/kg/day respectively [24], [25], [26].

### 4.3 Proximate analysis

Homogenized samples of a mixture of all samples were analyzed for moisture, protein, fat, fiber, and ash. Each analysis was carried out in duplicate according to the Association of Official Analytical Chemists standard procedure [27]. Moisture content was determined using the hot oven method [27]. 5 g of sample was dried in an oven at atmospheric pressure and at a temperature of 103 ± 2°C for 24 hours until a constant weight was obtained. Protein content was determined using Kjeldahl's method described by [27]. This method consists of first mineralizing the proteins with sulfuric acid to give ammonium sulfate. The sulfate is then distilled to produce ammonia gas, which is measured by titration. The percentage of crude protein was calculated by multiplying the nitrogen percentage by the protein factor of 6.25. Total fat in samples was determined using the hot Soxhlet extraction method [27] with diethyl ether solvent. Ash content was obtained after combustion in a muffle furnace at 550 °C until white ash was obtained (after 48 hours). Fiber content was also determined by the AOAC 2000 method. The samples were subjected to wet digestion by an acid, washed with boiled water, and wet digestion by a base again. The residue was collected and incinerated until a constant mass was obtained.

## 5 Results and discussion

### 5.1 Metals

The presence of potassium, sodium, magnesium, iron, nickel and chromium were all detected by the MP-AES and are presented in Table 1. Potassium is very important for regulating the normal electrical activity of the heart and low potassium intake has been linked to hypertension [28], [29]. Sodium is an essential mineral that regulates blood volume, blood pressure, osmotic equilibrium, and pH. High sodium consumption is unhealthy and can lead to chronic kidney disease, high blood pressure, cardiovascular diseases, and stroke [30], [31]. Magnesium is a cofactor which participates in the formation of the skeleton, muscle, soft tissues, and enhanced lipid profile in the body, in addition to enzyme modulation [32]. Its deficiency has been associated with impaired memory [33]. An essential element like Iron (Fe) is important for transporting oxygen and enzymes to other parts of the body. Iron forms complexes with molecular oxygen in hemoglobin and myoglobin, which play a key role in many important protein metabolisms that occur in the human body. Inadequate iron in the blood, also known as sideropenia, results in iron deficiency anemia, a condition that affects millions of people annually [34], fatigue thrombocytosis and many pregnancy and growth-related complications [35].

**Table 1:** Concentration of metals in Samples (mg/kg).

**Sample ID**	**[K]**	**[Na]**	**[Mg]**	**[Fe]**	**[Ni]**	**[Cr]**
1	3486.63	5710.00	321.00	117.00	6.17	13.80
2	236.24	5180.00	185.00	55.70	0.99	1.49
3	1127.15	9300.00	229.00	65.80	0.49	1.01
4	2550.69	4820.00	220.00	46.40	0.48	2.09
5	4896.85	7200.00	366.00	137.00	0.49	2.02
6	1846.01	8500.00	193.00	103.00	0.48	2.09
7	1269.38	5840.00	130.00	107.00	0.49	2.30
8	3319.62	7720.00	206.00	78.50	0.95	2.10
9	2863.43	7050.00	189.00	51.40	0.49	1.01
10	4268.52	6610.00	285.00	40.60	0.98	0.29
11	356.47	10200.00	252.00	95.40	3.46	0.11
12	319.93	16500.00	163.00	95.00	0.45	2.20
13	205.75	12400.00	223.00	115.00	0.49	2.03
14	101.05	12400.00	130.00	78.60	0.47	2.14
15	255.22	4080.00	306.00	67.40	0.48	2.08
16	130.34	12600.00	164.00	97.40	0.49	0.25
Maximum	4896.85	16500.00	366.00	137.00	6.17	13.80
Minimum	101.05	4080.00	130.00	40.60	0.45	0.11

**Fig. 1:**
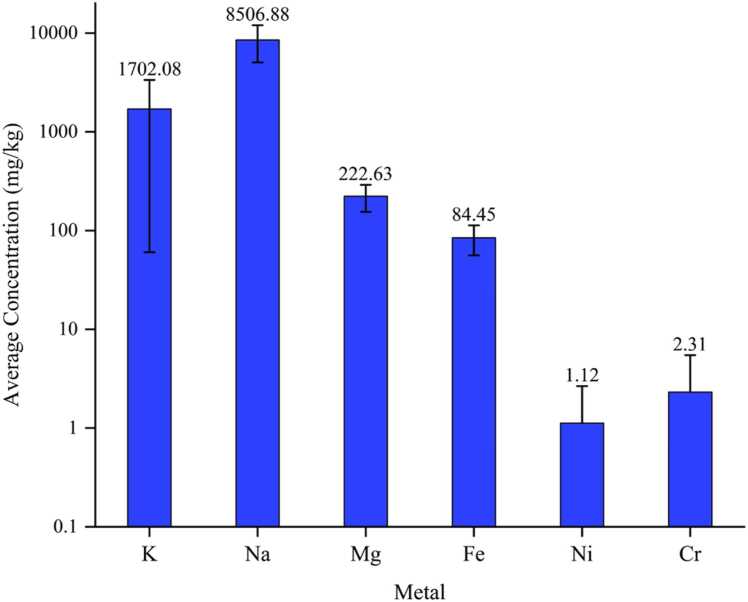
Average concentration of various metals in samples.

The results in this study indicate relatively high average concentration of potassium and sodium of 1702.08 mg/kg and 8506.88 mg/kg respectively with nickel having the lowest concentration of 1.12 mg/kg as shown in Fig. 1. Nickel concentration was higher than concentration measured for rice in southwestern Iran [36] and Hoveyzeh city [37].

The average concentration of potassium and sodium were found to be higher than that detected in yam peels obtained from yams purchased from Bosso market in Minna, Niger state in Nigeria by Lawal et al., 2014. It was reported that the average concentrations of sodium and potassium were 99.50 mg/100 g and 137.00 mg/100 g respectively. Iron levels were however lower in this study compared to the 68.5 mg/100 g of iron reported [38]. The relatively higher concentration of sodium in wasawasa may be attributed to the addition of common salt during the preparation of wasawasa. Common salt is primarily sodium chloride, therefore loading the wasawasa with extra sodium.

The pairwise relationships within the data were found using Pearson's correlation analysis, and Table 2 displays the findings. There were strong positive associations (r >0.7) between the Cr/Ni (0.79) values. There was a moderate negative association (r = −0.4 to −0.7) between Na/K (−0.52) and a moderate positive correlation (r = 0.4–0.7) between Mg/K (0.59). The correlations between the pairs of metals may be due to their common origin.

**Table 2:** Metal-to-metal correlation matrix for samples (r = 95 %).

	K	Na	Mg	Fe	Ni	Cr
K	1.00					
Na	**−0.52**^a^	1.00				
Mg	**0.59**^a^	−0.45^b^	1.00			
Fe	−0.09	0.46^b^	0.10	1.00		
Ni	0.23	−0.22	0.43^b^	0.18	1.00	
Cr	0.29	−0.24	0.35	0.28	**0.79**^a^	1.00

a Correlation is significant at the 0.05 level (two tailed), P < 0.05.

b Correlation is not significant at the 0.05 level (two tailed), P > 0.05.

The amount of metals involved when an adult with an average weight of 70 kg or a child with an average weight of 15 kg consumes 0.1 kg or 0.2 kg of wasawasa daily was used to calculate the estimated daily intake (EDI) of the metals in the groundnut paste samples. The Permitted Maximum Daily Intake (PMTDI) values suggested by the US Environmental Protection Agency (USEPA) were compared to the EDI values produced and shown in Table 3 and Table 4.

**Table 3:** Estimated daily intake of metals for adults (mg/kg/day).

**Sample ID**	**K**	**Na**	**Mg**	**Fe**	**Ni**	**Cr**
1	4.981	8.157	0.459	0.167	0.009	0.020
2	0.337	7.400	0.264	0.080	0.001	0.002
3	1.610	13.286	0.327	0.094	0.001	0.001
4	3.644	6.886	0.314	0.066	0.001	0.003
5	6.996	10.286	0.523	0.196	0.001	0.003
6	2.637	12.143	0.276	0.147	0.001	0.003
7	1.813	8.343	0.186	0.153	0.001	0.003
8	4.742	11.029	0.294	0.112	0.001	0.003
9	4.091	10.071	0.270	0.073	0.001	0.001
10	6.098	9.443	0.407	0.058	0.001	0.000
11	0.509	14.571	0.360	0.136	0.005	0.000
12	0.457	23.571	0.233	0.136	0.001	0.003
13	0.294	17.714	0.319	0.164	0.001	0.003
14	0.144	17.714	0.186	0.112	0.001	0.003
15	0.365	5.829	0.437	0.096	0.001	0.003
16	0.186	18.000	0.234	0.139	0.001	0.000
**Average**	**2.432**	**12.153**	**0.318**	**0.121**	**0.002**	**0.003**
**PMTDI**				**0.700**	**0.02**	**0.003**

**Table 4:** Estimated daily intake of metals for Children (mg/kg/day).

**Sample ID**	**K**	**Na**	**Mg**	**Fe**	**Ni**	**Cr**
**1**	46.488	76.133	4.280	1.560	0.082	0.184
**2**	3.150	69.067	2.467	0.743	0.013	0.020
**3**	15.029	124.000	3.053	0.877	0.007	0.013
**4**	34.009	64.267	2.933	0.619	0.006	0.028
**5**	65.291	96.000	4.880	1.827	0.007	0.027
**6**	24.613	113.333	2.573	1.373	0.006	0.028
**7**	16.925	77.867	1.733	1.427	0.007	0.031
**8**	44.262	102.933	2.747	1.047	0.013	0.028
**9**	38.179	94.000	2.520	0.685	0.007	0.013
**10**	56.914	88.133	3.800	0.541	0.013	0.004
**11**	4.753	136.000	3.360	1.272	0.046	0.001
**12**	4.266	220.000	2.173	1.267	0.006	0.029
**13**	2.743	165.333	2.973	1.533	0.007	0.027
**14**	1.347	165.333	1.733	1.048	0.006	0.029
**15**	3.403	54.400	4.080	0.899	0.006	0.028
**16**	1.738	168.000	2.187	1.299	0.007	0.003
**Average**	**22.694**	**113.425**	**2.968**	**1.126**	**0.015**	**0.031**
**PMTDI**				**0.700**	**0.020**	**0.003**

The average EDI trend was Na > K > Mg > Fe > Cr > Ni. The concentrations of elements and amount ingested in a day, when compared to the available values were generally lower than the permitted maximum daily intake for adults whereas the average EDIs for children were generally higher than the USEPA limits except for nickel intake.

### 5.2 Hazards quotients and index

Hazard quotient (HQ) calculations were carried out to show the likelihood of an individual experiencing an effect associated with metal contamination. The Hazard Quotient (HQ) values as presented on Table 5 were found to be greater than one for chromium among adults and children, and iron for children, whereas HQ values less than one for nickel among adults and children and iron in adults was obtained. Health risk analysis indicated the a likelihood of potential health hazard especially from potential bioaccumulation [39]. Similar results were obtained for milled shrimps in Kumasi [20].

**Table 5:** Hazard Quotients and Hazard index.

**Sample ID**	**Fe**		**Ni**		**Cr**		**HI**	
	**Children**	**Adult**	**Children**	**Adult**	**Children**	**Adult**	**Children**	**Adult**
**1**	2.23	0.24	4.11	0.44	61.33	6.57	67.68	7.25
**2**	1.06	0.11	0.66	0.07	6.62	0.71	8.35	0.89
**3**	1.25	0.13	0.33	0.04	4.49	0.48	6.07	0.65
**4**	0.88	0.09	0.32	0.03	9.29	1.00	10.49	1.12
**5**	2.61	0.28	0.33	0.04	8.98	0.96	11.92	1.28
**6**	1.96	0.21	0.32	0.03	9.29	1.00	11.57	1.24
**7**	2.04	0.22	0.33	0.04	10.22	1.10	12.59	1.35
**8**	1.50	0.16	0.63	0.07	9.33	1.00	11.46	1.23
**9**	0.98	0.10	0.33	0.04	4.49	0.48	5.80	0.62
**10**	0.77	0.08	0.65	0.07	1.29	0.14	2.72	0.29
**11**	1.82	0.19	2.31	0.25	0.49	0.05	4.62	0.49
**12**	1.81	0.19	0.30	0.03	9.78	1.05	11.89	1.27
**13**	2.19	0.23	0.33	0.04	9.02	0.97	11.54	1.24
**14**	1.50	0.16	0.31	0.03	9.51	1.02	11.32	1.21
**15**	1.28	0.14	0.32	0.03	9.24	0.99	10.85	1.16
**16**	1.86	0.20	0.33	0.04	1.12	0.12	3.31	0.35
**Average**	**1.61**	**0.17**	**0.74**	**0.08**	**10.28**	**1.10**	**12.63**	**1.35**

The Hazard Index (HI) was determined by summing up the Hazard Quotients for the individual metals, which indicated a likelihood of potential health hazard.

Table 6 presents the results of proximate analysis compared with yam constituent values obtained from the Ghana Center for Scientific and Industrial Research (CSIR). The largest constituent of wasawasa was moisture, which makes up 27.54 percent of the mass. The moisture content was however lower than reported values for the yam varieties reported by CSIR. This indicates that wasawasa, which is made from yam peels has lower moisture content compared to the flesh of different yam varieties. The protein and fat content in wasawasa was higher than the amounts found in the flesh of the different varieties. Conversely the fiber and ash contents were generally lower in wasawasa. When compared to proximate analysis of yam peel by Lawal et al., 2014, the protein and moisture content in this study was higher than the protein (3.46 m/100 g) and moisture (11.75 g/100 g) reported [38].

**Table 6:** Proximate analysis.

**Sample ID**	**Protein**	**moisture**	**fats**	**fiber**	**Ash**
**Mixture**	12.81	27.54	0.45	0.41	1.00
**Kpuna**	3.30	51.60	0.18	1.83	1.40
**Laabako**	1.70	52.60	0.20	2.09	
**Zongo**	2.20	61.00	0.26	2.19	1.60
**Manchisi**	1.60	71.90	0.26	1.96	1.00
**Chamba**	3.20	50.20	0.23	2.15	1.00
**Kangbringa**	4.20	57.80	0.16	2.20	1.20

## 6 Conclusion

The results indicated relatively high protein content of wasawasa which may be important for meeting the protein needs of consumers. The presence of metals (Fe, Na, Ni, Cr, Mg, and K) in wasawasa with varied concentrations were also detected and their computed risk analysis suggested a likelihood of experiencing an effect associated with the heavy metal (Fe, Ni, and Cr) contamination especially with bioaccumulation. It is recommended that vendors be educated in terms of minimizing the amount of salt added to the dish to minimize the sodium content in the food and there should be public education on the health risk associated with elevated metal content in wasawasa.

## Ethical approval

Ethical clearance was not required for this study as no human participants were involved.

## Funding

This study did not receive any funding.

## Author Statement

We the above listed authors declare that this manuscript is original, has not been published before and is not currently being considered for publication elsewhere. We confirm that the manuscript has been read and approved by all named authors and that there are no other persons who satisfied the criteria for authorship but are not listed. We further confirm that the order of authors listed in the manuscript has been approved by all of us. We understand that the Corresponding Author is the sole contact for the Editorial process. She is responsible for communicating with the other authors about progress, submissions of revisions and final approval of proofs

## CRediT authorship contribution statement

**Dominic Adrewie:** Writing – original draft, Validation, Formal analysis. **Fati Haruna:** Writing – original draft, Investigation, Formal analysis, Data curation. **Marian Asantewah Nkansah:** Writing – review & editing, Supervision, Conceptualization.

## Declaration of Competing Interest

The authors certify that they have NO affiliations with or involvement in any organization or entity with any financial or non-financial interests in the subject matter or materials discussed in this manuscript.

## Data Availability

All data associated with the article have been shared
